# Niemann-Pick Type C2 Protein Mediates Hepatic Stellate Cells Activation by Regulating Free Cholesterol Accumulation

**DOI:** 10.3390/ijms17071122

**Published:** 2016-07-13

**Authors:** Yuh-Ching Twu, Tzong-Shyuan Lee, Yun-Lian Lin, Shih-Ming Hsu, Yuan-Hsi Wang, Chia-Yu Liao, Chung-Kwe Wang, Yu-Chih Liang, Yi-Jen Liao

**Affiliations:** 1 Department of Biotechnology and Laboratory Science in Medicine, School of Biomedical Science and Engineering, National Yang-Ming University, 112 Taipei, Taiwan; yctwu@ym.edu.tw; 2 Department & Institute of Physiology, National Yang-Ming University, 112 Taipei, Taiwan; tslee@ym.edu.tw; 3 College of Biopharmaceutical and Food Sciences, China Medical University, 404 Taichung, Taiwan; yllin@mail.cmu.edu.tw; 4 Department of Biomedical Imaging and Radiological Sciences, National Yang-Ming University, 112 Taipei, Taiwan; smhsu@ym.edu.tw; 5 School of Medical Laboratory Science and Biotechnology, College of Medical Science and Technology, Taipei Medical University, 110 Taipei, Taiwan; sunnyw0228@gmail.com (Y.-H.W.); fish88094@yahoo.com.tw (C.-Y.L.); ycliang@tmu.edu.tw (Y.-C.L.); 6 Department of International Medicine, Taipei City Hospital Ranai Branch, 106 Taipei, Taiwan; ckwang88@ms74.hinet.net

**Keywords:** Niemann-Pick type C2, hepatic stellate cells, free cholesterol, transforming growth factor-β1, liver fibrosis

## Abstract

In chronic liver diseases, regardless of their etiology, the development of fibrosis is the first step toward the progression to cirrhosis, portal hypertension, and hepatocellular carcinoma. Hepatic stellate cells (HSCs) are the main profibrogenic cells that promote the pathogenesis of liver fibrosis, and so it is important to identify the molecules that regulate HSCs activation and liver fibrosis. Niemann-Pick type C2 (NPC2) protein plays an important role in the regulation of intracellular cholesterol homeostasis by directly binding with free cholesterol. However, the roles of NPC2 in HSCs activation and liver fibrosis have not been explored in detail. Since a high-cholesterol diet exacerbates liver fibrosis progression in both rodents and humans, we propose that the expression of NPC2 affects free cholesterol metabolism and regulates HSCs activation. In this study, we found that NPC2 is decreased in both thioacetamide- and carbon tetrachloride-induced liver fibrosis tissues. In addition, NPC2 is expressed in quiescent HSCs, but its activation status is down-regulated. Knockdown of NPC2 in HSC-T6 cells resulted in marked increases in transforming growth factor-β1 (TGF-β1)-induced collagen type 1 α1 (Col1a1), α-smooth muscle actin (α-SMA) expression, and Smad2 phosphorylation. In contrast, NPC2 overexpression decreased TGF-β1-induced HSCs activation. We further demonstrated that NPC2 deficiency significantly increased the accumulation of free cholesterol in HSCs, increasing Col1a1 and α-SMA expression and activating Smad2, and leading to sensitization of HSCs to TGF-β1 activation. In contrast, overexpression of NPC2 decreased U18666A-induced free cholesterol accumulation and inhibited the subsequent HSCs activation. In conclusion, our study has demonstrated that NPC2 plays an important role in HSCs activation by regulating the accumulation of free cholesterol. NPC2 overexpression may thus represent a new treatment strategy for liver fibrosis.

## 1. Introduction

Liver cirrhosis is a major cause of mortality and liver transplantation worldwide, and its therapeutic options are limited. In chronic liver diseases, regardless of their etiology (e.g., viral infection, alcohol abuse, or non-alcoholic fatty liver), the development of fibrosis is the first step toward the progression to cirrhosis, portal hypertension and hepatocellular carcinoma (HCC) [1,2,3]. Liver fibrosis is characterized by excessive deposition of extracellular matrix (ECM) and fibrous scar formation [4]. Hepatic stellate cells (HSCs) are the major profibrogenic cells that produce ECM proteins in the damaged liver [5]. In response to injurious agents or exposure to inflammatory cytokines, HSCs undergo an activation process from a fat-storing quiescent phenotype to a myofibroblastic phenotype with significantly increased proliferation, chemotaxis, fibrogenesis, contractility, ECM degradation and retinoid loss [6,7,8,9]. Transforming growth factor-β1 (TGF-β1) is recognized as the key cytokine that activates HSCs and directly induces the expression of collagen 1 and α-smooth muscle actin (α-SMA) via the Smad2/3 signaling pathway, thus causing hepatic fibrogenesis and cancer progression [10,11,12]. Antagonism of TGF-β1 signaling pathways markedly decreases fibrosis [13,14].

Niemann-Pick type C2 (NPC2) is a small soluble glycoprotein that is mainly expressed in the liver, kidney, and testis [15,16]. NPC2 plays an important role in regulating intracellular cholesterol trafficking and homeostasis by directly binding with free cholesterol [17,18]. The mature human NPC2 protein comprises 132 amino acids and is expressed in different isoforms that vary in size from 19 to 23 kDa in a tissue-specific manner [15,19]. A deficiency in NPC2 results in the accumulation of free cholesterol in the lysosome [19,20]. We recently reported that NPC2 down-regulation is correlated with the α-fetoprotein level, tumor type, vascular invasion, and pathology stages of HCC patients. In-vitro and in vivo xenograft data demonstrated that the expression of NPC2 regulates cell proliferation, migration, and tumorigenesis by regulating ERK1/2 activation [21]. Previously, we conducted an immunohistochemical-based study to examine the expression of NPC2 in a variety of different human cancers, and meanwhile NPC2 was found to be down-regulated in human liver cirrhosis and hepatoma tissues [22]. However, the molecular mechanisms of NPC2 in HSCs activation and liver fibrosis have not been explored in detail.

Both unadjusted and adjusted analyses have shown that higher dietary consumption of cholesterol in humans is associated with a higher risk of cirrhosis or liver cancer [23] Cholesterol-lowering agents improve liver fibrosis in patients with hypercholesterolemia [24]. Several recent in vivo and in vitro studies found that a high-cholesterol diet exacerbates carbon tetrachloride (CCl_4_) and bile duct ligation-induced liver fibrosis in rodents [25]. The breakdown of intracellular lipid droplets provides a key source of energy for fueling HSC activation [26]. The free cholesterol accumulation increases the sensitization of HSCs to TGF-β1 and plays an important role in the pathogenesis of liver fibrosis [25,27,28]. Accordingly, we hypothesized that the expression of NPC2 mediates free cholesterol accumulation and regulates HSCs activation and liver fibrosis.

In this study we show that NPC2 is down-regulated in CCl_4_- and thioacetamide (TAA)-induced liver fibrosis tissues. The loss of NPC2 enhanced the accumulation of free cholesterol in HSCs and made them more susceptible to TGF-β1 treatment. In contrast, NPC2 overexpression diminished free cholesterol accumulation and attenuated TGF-β1-induced HSCs activation.

## 2. Results

### 2.1. NPC2 Is down-Regulated in Liver Fibrosis Tissues

**Figure 1:**
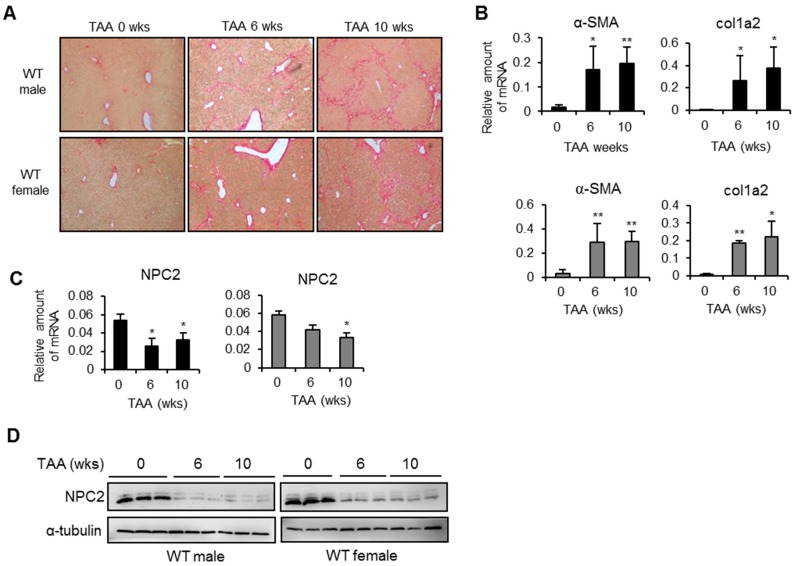
Niemann-Pick type C2 (NPC2) is down-regulated in thioacetamide (TAA)-induced liver fibrosis tissues. (**A**) Representative sirius red staining images of liver tissues from mice treated with TAA for 0, 6, and 10 weeks. Original magnification, ×100; (**B**,**C**) Results from Q-PCR analyses of α-smooth muscle actin (α-SMA), collagen type 1 α2 (Col1a2), and NPC2 gene expression in TAA-treated wild-type (WT) male (black bar) and WT female (gray bar) mice; (**D**) Representative results from Western blot analyses of NPC2 expression in TAA-treated WT mice. *n* = 5 per group. Data are shown as mean ± SD. * *p* < 0.05; ** *p* < 0.01 vs. no TAA treatment.

NPC2 is strongly expressed in normal liver tissues, but is down-regulated in HCC tissues [21]. To determine whether NPC2 expression is associated with liver fibrosis, we used the mouse model in which liver fibrosis is produced by the i.p. injection of TAA. As shown in Figure 1A, sirius red staining demonstrated fibrillar collagen deposition in the livers of TAA-treated wild-type (WT) mice. The Q-PCR analyses showed that the mRNA levels of the liver fibrosis marker genes (α-SMA and Col1a2) were up-regulated in both male and female WT mice (Figure 1B), while hepatic NPC2 was decreased (Figure 1C). The protein levels of NPC2 were down-regulated in WT mice of both sexes after 6 and 10 weeks of TAA treatment. (Figure 1D).

**Figure 2:**
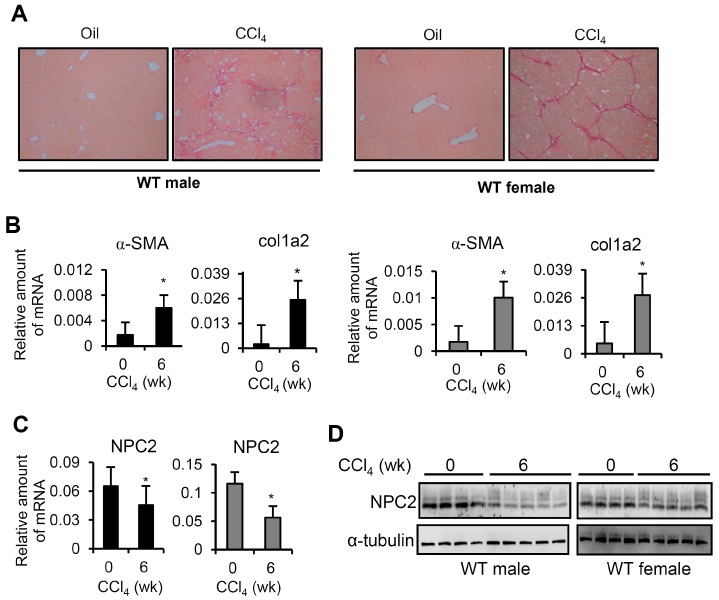
NPC2 is down-regulated in CCl_4_-induced liver fibrosis tissues. (**A**) Representative sirius red staining images of liver tissues from mice treated with CCl_4_ for 0 and 6 weeks. Original magnification, ×100; (**B**,**C**) Results from Q-PCR analyses of α-SMA, Col1a2 and NPC2 gene expression in CCl_4_-treated WT male (black bar) and WT female (gray bar) mice; (**D**) Representative results from Western blot analyses of NPC2 expression in CCl_4_-treated WT mice. *n* = 5 per group. Data are shown as mean ± SD. * *p* < 0.05 vs. no CCl_4_ treatment.

In a similar manner to the TAA treatment, the CCl_4_-induced model of liver fibrosis showed a significant fibrillar collagen deposition in WT mice of both sexes (Figure 2A). The mRNA expression levels of α-SMA and Col1a2 were significantly increased after the development of CCl_4_-induced liver fibrosis (Figure 2B). The hepatic mRNA and protein levels of NPC2 were down-regulated in WT mice of both sexes after 6 weeks of CCl_4_ treatment (Figure 2C,D). These data imply that liver fibrosis had been successfully induced and that hepatic NPC2 was down-regulated in WT mice of both sexes.

### 2.2. NPC2 Is Expressed in HSCs but Is down-Regulated in Activated HSCs

**Figure 3:**
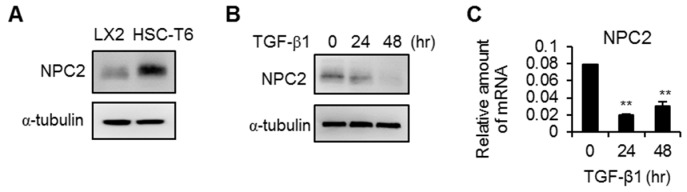
NPC2 is expressed in hepatic stellate cells (HSCs) and is down-regulated in activated HSCs. (**A**) Results from Western blot analyses of NPC2 expression in quiescent LX2 and HSC-T6 cells; (**B**,**C**) Western blot (**B**) and Q-PCR (**C**) analyses of NPC2 expression in LX2 cells treated with 20 ng/mL transforming growth factor-β1 (TGF-β1). Data are expressed as mean ± SD. ** *p* < 0.01 vs. time 0.

Since HSCs are the main mediator of fibrogenesis, we next examined whether NPC2 is expressed in HSCs. As shown in Figure 3A, NPC2 could be detected in both LX2 and HSC-T6 cells. Since TGF-β1 is the most potent factor for HSCs activation and human fibrogenesis [11,12], we treated the LX2 cells with TGF-β1 to activate HSCs and then measured the expression of NPC2. As shown in Figure 3B,C, the protein and mRNA levels of NPC2 were down-regulated in TGF-β1-treated LX2 cells.

### 2.3. Knockdown of NPC2 in HSCs Increases TGF-β1-Induced Fibrogenesis

**Figure 4:**
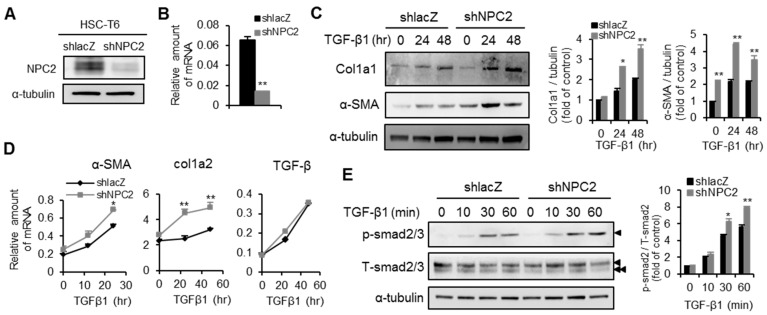
Knockdown of NPC2 increases TGF-β1induced fibrogenesis in HSC-T6 cells. HSC-T6 cells were infected by lentiviruses carrying the shlacZ control and shNPC2 gene. After puromycin-based selection for 1 week, the cells were harvested and confirmed by (**A**) Western blotting and (**B**) Q-PCR; (**C**) Different lentiviruses infected HSC-T6 stable cells were treated with 20 ng/mL TGF-β1 for the indicated time periods, and the lysates were immunoblotted and then quentified to detect α-SMA and Col1a1; (**D**) Results from Q-PCR analyses of α-SMA, Col1a2 and TGF-β expression in HSC-T6 stable cells treated with 20 ng/mL TGF-β1. * *p* < 0.05; ** *p* < 0.01 vs. shlacZ control; (**E**) Different HSC-T6 stable cells were treated with 20 ng/mL TGF-β1 for the indicated time periods, and the lysates were subjected to Western blot analysis and quentification to detect Smad2 phosphorylation (►, Smad2, ►►, Smad3). Each experiment was performed in three independent replicates. A similar phenomenon was observed. Therefore, the representative data was shown in the figure.

To elucidate the roles of NPC2 down-regulation in the activation of HSCs, the knock-down strategy was applied in HSC-T6 cells by infecting them with lentivirus carrying shRNA targeted at NPC2. As shown in Figure 4A,B, the protein and mRNA levels of NPC2 were decreased relative to the shlacZ control. Regarding the effects of NPC2 expression on HSCs activation, the expressions levels of Col1a1 and α-SMA were higher in TGF-β1-treated shNPC2 cells (Figure 4C). Consistent with the protein levels, knockdown of NPC2 resulted in markedly increased expression of both α-SMA and Col1a2 mRNAs, whereas the expression of TGF-β did not differ from that in shlacZ control (Figure 4D). Once TGF-β1 binds to its receptor, Smad2/3 protein will be phosphorylated and then trigger collagen production [9,11]. Therefore, we tested whether down-regulation of NPC2 influences smad2/3 activation in HSCs treated with TGF-β1. As shown in Figure 4E, shNPC2 enhanced the phosphorylation of Smad2relative to the shlacZ control.

### 2.4. NPC2 Overexpression Attenuates TGF-β1-Induced Fibrogenesis

**Figure 5:**
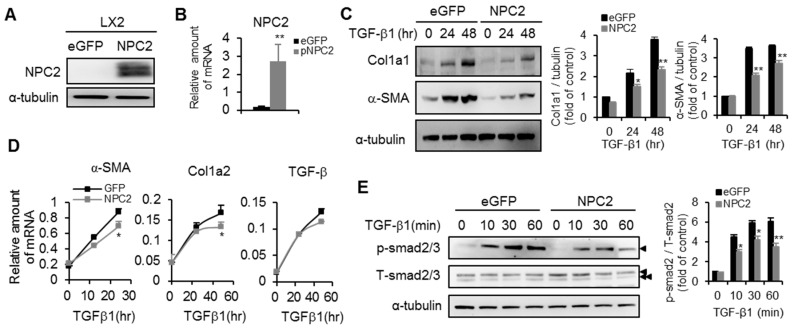
Overexpression of NPC2 attenuates TGF-β1-induced fibrogenesis in LX2 cells. LX2 cells were infected by lentiviruses carrying the eGFP control and NPC2 gene. After puromycin-based selection for 1 week, the cells were harvested and confirmed by (**A**) Western blotting and (**B**) Q-PCR; (**C**) Different lentiviruses infected LX2 stable cells were treated with 20 ng/mL TGF-β1for the indicated time periods, and the lysates were immunoblotted and then quentified to detect α-SMA and Col1a1; (**D**) Results from Q-PCR analyses of α-SMA, Col1a2 and TGF-β expression in LX2 stable cells treated with 20 ng/mL TGF-β1. * *p* < 0.05; ** *p* < 0.01 vs. eGFP control; (**E**) Different LX2 stable cells were treated with 20 ng/mL TGF-β1 for the indicated time periods, and the lysates were immunoblotted and then quentified to detect Smad2 phosphorylation (►, smad2, ►►, smad3). Each experiment was performed in three independent replicates. A similar phenomenon was observed. Therefore, the representative data was shown in the figure.

We next used an overexpression approach to delineate the roles of NPC2 in the inhibition of HSCs activation. The LX2 cells were infected with lentivirus carrying the NPC2 gene, and the results showed that NPC2 expression was significantly increased at both the protein and mRNA levels (Figure 5A,B). Regarding TGF-β1-induced fibrogenesis in HSCs, NPC2 overexpression significantly decreased the expression levels of Col1a1 and α-SMA (Figure 5C,D), while, there was no difference in TGF-β expression between LX2 cells with eGFP and NPC2 (Figure 5D). The phosphorylation of Smad2 was diminished in NPC2 overexpressed LX2 cells relative to the eGFP control cells (Figure 5E).

### 2.5. NPC2 down Regulation Induced Free Cholesterol Accumulation Sensitizes HSCs to TGF-β1 Treatment

**Figure 6:**
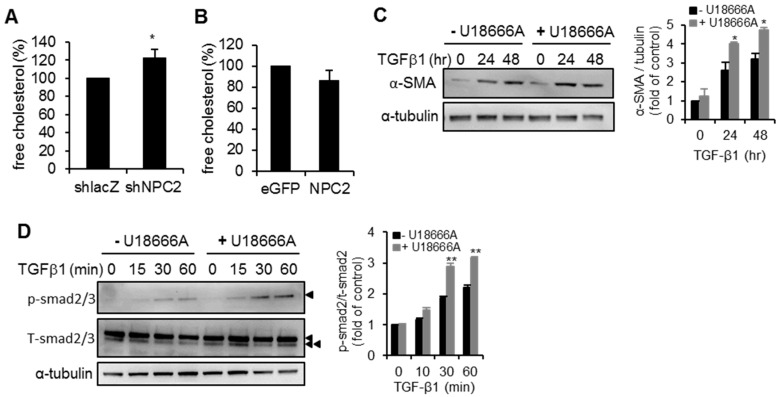
NPC2 down-regulation induced free cholesterol accumulation sensitizes HSCs to TGF-β1 treatment. (**A**,**B**) Quantification and comparison of the percentages of intracellular free cholesterol in different lentiviruses infected stable cells. * *p* < 0.05 vs. shlacZ control; (**C**,**D**) LX2 cells were pretreated with 1 µM U18666A-overnight to induce free cholesterol accumulation and then treated with 20 ng/mL TGF-β1. Cell lysates were immunoblotted and then quentified to detect α-SMA and Smad2 phosphorylation (►, Smad2, ►►, Smad3). ** *p* < 0.01 vs. – U18666A. Representative blots of three independent experiments with similar results are shown.

Previous studies have highlighted the abnormal accumulation of free cholesterol in NPC2 mutant cells [17,19]. We therefore compared the intracellular free cholesterol levels between shlacZ and shNPC2 in HSC-T6 cells, and between eGFP and NPC2 in LX2 cells. As shown in Figure 6A, the level of free cholesterol was significantly higher in the NPC2 knock-down cells than in the shlacZ control cells. However, no difference was noted between eGFP control and NPC2 overexpressed cells (Figure 6B). Given that the accumulation of free cholesterol is associated with the loss of NPC2 expression, we sought to determine whether a similar phenotype exists following treatment with U18666A (an inhibitor of cholesterol transport), which is a positive control to induce free cholesterol accumulation. We pretreated LX2 cells with or without U18666A overnight and then applied TGF-β1 treatment. As shown in Figure 6C, U18666A treatment accelerated the α-SMA expression after 24 h. In addition, the phosphorylation of Smad2 was significantly increased in U18666A-pretreated cells (Figure 6D).

### 2.6. NPC2 Overexpression Decreases U18666A- and TGF-β1-Induced Free Cholesterol Accumulation and HSCs Activation

**Figure 7:**
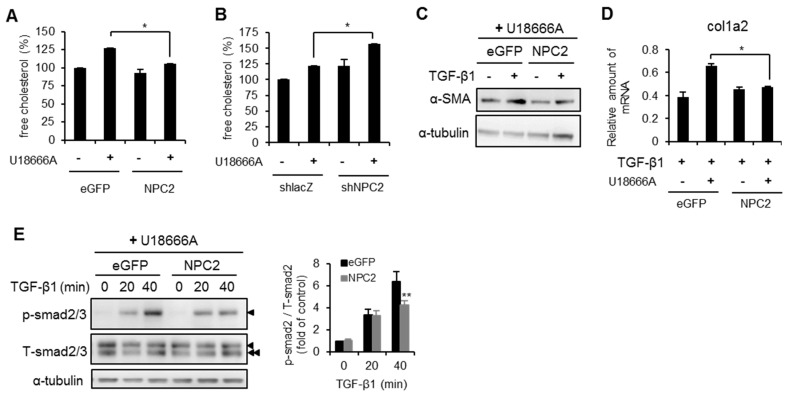
Overexpression of NPC2 diminishes U18666A- and TGF-β1-induced free cholesterol accumulation and HSCs activation. (**A**,**B**) Different lentiviruses infected stable cells were pretreated with or without 1 µM U18666A overnight and then subjected to intracellular free cholesterol quantification; (**C**–**E**) eGFP- and NPC2-overexpressed LX2 cells were pretreated with 1 µM U18666A overnight and then treated with 20 ng/mL TGF-β1. Cell lysates were immunoblotted to detect α-SMA (**C**) and Smad2 phosphorylation (►, Smad2, ►►, Smad3) (**E**). mRNA expression of Col1a2 was analyzed using Q-PCR (**D**). * *p* < 0.05. ** *p* < 0.01 vs. eGFP. Each experiment was performed in three independent replicates. A similar phenomenon was observed. Therefore, the representative data was shown in the figure.

Finally, we evaluated whether NPC2 overexpression can attenuate U18666A-induced free cholesterol accumulation and reduce HSCs activation. We first determined the level of free cholesterol in U18666A-treated HSCs. As shown in Figure 7A, overexpression of NPC2 decreased the U18666A-induced free cholesterol accumulation relative to the eGFP control. In contrast, NPC2 loss increased the U18666A-induced free cholesterol accumulation relative to the shlacZ control (Figure 7B). Since the accumulation of free cholesterol in HSCs plays an important role in the progression of liver fibrosis and sensitizing HSCs to TGF-β1-induced activation [25,28], we tested whether overexpression of NPC2 decreases TGF-β1-induced HSCs activation under the condition of free cholesterol accumulation. As compared with eGFP control, NPC2 overexpression significantly reduced α-SMA and Col1a2 expression (Figure 7C,D). After applying U18666A pretreatment overnight, the TGF-β1-induced phosphorylation of Smad2 was significantly reduced in NPC2-overexpressed cells (Figure 7E).

## 3. Discussion

NPC2 was first characterized as a major secretory protein in human epididymis to function as a key regulator in free cholesterol homeostasis [17,29]. We have previously provided evidence that down-regulation of NPC2 is correlated with clinicopathological features and regulates ERK1/2 activation in liver cancer [21]. However, the pathophysiological role of NPC2 in HSCs activation and liver fibrosis has not been defined previously. In the present study we found that NPC2 was down-regulated in both TAA- and CCl_4_-induced liver fibrosis tissues. In addition, NPC2 was expressed in quiescent HSCs, but its expression level was decreased in TGF-β1-treated HSCs. Knockdown of NPC2 in HSCs increased the accumulation of free cholesterol, which led to the sensitization of HSCs to TGF-β1 exposure. In contrast, overexpression of NPC2 decreased the free cholesterol accumulation and the TGF-β1-induced HSCs activation. Our results support a novel role of NPC2 in HSCs activation, in that NPC2-dependent regulation is crucial for modulating the metabolism of free cholesterol and for activating HSCs during liver fibrosis.

The liver plays a critical role in whole-body cholesterol metabolism [30,31]. A higher dietary consumption of cholesterol is associated with a higher risk of cirrhosis and liver cancer [23]. Intracellular cholesterol homeostasis is maintained through de novo biosynthesis and low-density lipoprotein receptor (LDLR) pathway salvage [32,33]. Regarding the salvaging of extracellular cholesterol, low-density lipoprotein carrying cholesterol and cholesterol esters bound to LDLR is internalized and transported to sorting endosomes and then subsequently to late endosomes and lysosomes from which cholesterol esters are hydrolyzed to free cholesterol by acid lipase [30]. NPC2 is a lysosomal protein that binds free cholesterol and transports to the endoplasmic reticulum where acyl-coenzyme A: cholesterol acetyltransferase (ACAT) esterification of free cholesterol for storage in lipid droplets [32,33]. Teratani et al. reported that a high-cholesterol diet exacerbates liver fibrosis in mice via the accumulation of free cholesterol in HSCs [25]. However, the reason for this accumulation of free cholesterol is not fully understood. In 2014, Tomita et al. reported that ACAT1 deficiency exaggerates liver fibrosis by increasing the level of free cholesterol in HSCs [27]. The present study has identified another molecule, named NPC2, as a key regulator that prevents the accumulation of free cholesterol in HSCs and decreases TGF-β1-induced HSCs activation. In addition, NPC2 down-regulation enhances free cholesterol accumulation in HSCs and makes these cells more susceptible to TGF-β1 exposure. Free cholesterol accumulation has been reported to increase TLR4 level through suppression of the endosomal-lysosomal degradation pathway of TLR4, and thereby sensitized the cells of TGF-β-induced activation [28]. In this study, we found that NPC2 is decreased in CCl_4_- or TAA-induced liver fibrosis tissue, as well as in TGF-β1-treated HSCs, however the regulation mechanism of NPC2 is not clear. Since Nogo-B receptor (NgBR) can interact with and stabilize the NPC2 [34], further studies are needed to elucidate whether NgBR regulates NPC2 level n in the pathogenesis of liver fibrosis.

We recently showed that NPC2 down-regulation in liver tumors is correlated with poor clinicopathological features [21]. In addition, NPC2 down-regulation enhanced the proliferation of hepatocytes and xenograft tumorigenesis via the activation of ERK1/2. U18666A treatment also demonstrated that inhibiting the transport of free cholesterol did not alter the proliferation and MAPK/ERK activation in hepatocytes [21]. Accordingly, our data indicate that NPC2 regulates ERK1/2 activation under a free cholesterol-independent condition especially in hepatocytes. Tomita et al. [28] and Teratani et al. [25] reported that a high cholesterol diet neither affected hepatocyte injury nor influenced the activation of Kupffer cells. Instead, the free cholesterol accumulation in HSCs increased and further sensitized these cells to TGF-β1-induced activation in a vicious cycle. Mari et al. found that an overload of free cholesterol was responsible for hepatocellular sensitivity to TNF-α induced apoptosis [35]. Nonetheless, several other studies found that TNF-α-mediated apoptosis was not involved in the progression of liver fibrosis [36,37]. Together these results suggest that HSCs, rather than hepatocytes, should serve as a major cause of alterations in liver fibrosis resulting from NPC2 down-regulation-induced free cholesterol accumulation.

In summary, our study has provided new insights into the mechanisms linking free cholesterol accumulation and HSCs activation through the expression of NPC2. Regulating of NPC2 in HSCs may therefore represent a new treatment strategy for liver fibrosis.

## 4. Materials and Methods

### 4.1. Animal Models

Eight-week-old C57BL/6 wild-type (WT) mice were purchased from the National Laboratory Animal Center, Taiwan. All mice were maintained on a standard chow diet (No. 5001, LabDiet, St. Louis, MO, USA) and housed in a 12-h/12-h light/dark cycle. To produce the CCl_4_-induced liver fibrosis model, the mice received an intraperitoneal (i.p.) injection of CCl_4_ (2 mL/kg body weight (1:5 *v*/*v* in mineral oil)) twice weekly for 6 weeks. In the TAA-induced liver fibrosis model, the mice received an i.p. injection of TAA (200 mg/kg body weight; Sigma-Aldrich, St. Louis, MO, USA) three times weekly for 6 and 10 weeks. The experimental protocols were approved by the Institutional Animal Care and Use Committee of Taipei Medical University. Liver samples were collected at the end of the experiments. The tissues used in protein and RNA analyses were frozen in liquid nitrogen and stored at −80 °C before use, while those used in IHC staining were fixed in 10% formalin.

### 4.2. Cell Culture, Plasmids and Lentiviral Infection

293T cells (ATCC No. CRL-11268) were cultured in Dulbecco’s modified Eagle’s medium (DMEM) (Gibco BRL, Grand Island, NY, USA) with 10% heat-inactivated fetal bovine serum (HyClone, Logan, UT, USA), penicillin (100 U/mL), streptomycin (100 μg/mL), nonessential amino acids (0.1 mM), and l-glutamine (2 mM) in a humidified incubator with 5% CO_2_. Plasmid encoding shRNA for NPC2 (pLKO.1-shNPC2) and control plasmids for the RNA interference experiments (pLKO.1-shlacZ) and for the overexpression experiments (pLKO_AS3w.eGFP.puro) were obtained from the National RNAi Core Facility (Academia Sinica, Taipei, Taiwan). 293T cells were co-transfected with a packaging plasmid-pCMV-ΔR8.91, a VSV-G envelope expressing plasmid-pMD.G and one of four lentiviral constructs (pLKO.1-shlacZ, pLKO.1-shNPC2, pLKO_AS3w.eGFP.puro or pLV-NPC2) using TurboFect^TM^ Reagent (Fermentas, Hanover, MD, USA). Supernatants containing lentiviruses were harvested according to the protocol published on the website http://rnai.genmed.sinica.edu.tw. To generate stable cell lines, LX2 cells (an immortalized strain of human HSCs) and HSC-T6 cells (an immortalized strain of rat HSCs) (as a gift from S. Friedman) [38] were infected with pseudo-typed lentivirus in a medium containing Polybrene (8 μg/mL). The cells were treated with 1 μg/mL puromycin at 24 h after infection to allow stable cells to be selected. Lentivirus-infected stable HSC-T6 cells containing: shlacZ and shNPC2, and LX2 cells containing eGFP and NPC2 were grown in DMEM supplemented with 1% heat-inactivated fetal bovine serum (FBS) and 1 μg/mL puromycin.

### 4.3. TGF-β1 and U18666A Treatment

To study the effect of NPC2 expression on TGF-β1-induced fibrogenesis, HSCs (growth in 1% heat-inactivated FBS) were seeded in 6 well plate (1 × 10^5^ per well) and treated with 20 ng/mL TGF-β1 (R&D Systems, Minneapolis, MN, USA) for different time periods. To induce the accumulation of free cholesterol, cells were pretreated with 1 µM U18666A [34] (Sigma-Aldrich) overnight and then treated with TGF-β1.

### 4.4. Western Blotting, IHC Staining, and Free Cholesterol Quantification

Cultured cells and liver tissues were lysed using lysis buffer supplemented with protease and phosphatase inhibitors. Proteins (30 µg) were separated by SDS-PAGE. Rabbit anti-p-Smad2/3 and T-Smad2/3 were purchased from Cell Signaling Technology (Beverly, MA, USA). Rabbit anti-α-SMA, Col1a1 and NPC2 were purchased from Abcam (Cambridge, MA, USA), and Santa Cruz Biotechnology (Santa Cruz, CA, USA), respectively. The immunoblotting signals of α-SMA and Col1a1 were normalized to that for α-tubulin (Sigma-Aldrich). The immunoblotting signals of p-smad2 was normalized to that for T-smad2 and quantified by densitometric scanning (ImageJ software, NIH, Bethesda, MD, USA). Sirius red staining (Abcam) of paraffin-embedded liver sections was used to qualitatively assess the collagen architecture and the extent of fibrosis in accordance with the manufacturer’s instructions. The intracellular concentration of free cholesterol was measured using a commercial colorimetric kit (BioVision, Mountain View, CA, USA).

### 4.5. Real-Time PCR

Total RNA was isolated from mouse liver using TRIzol Reagent (Ambion, Carlsbad, CA, USA), according to the manufacturer’s protocol. Complementary DNA was produced from cellular RNA (2 μg) using a SuperScript II RNase H-Reverse Transcriptase Kit (Invitrogen, Carlsbad, CA, USA). The following primers were used in the real-time PCR (Q-PCR): NPC2, 5′-CGGAGCCCCTGCACTTC-3′ (forward) and NPC2: 5′-GGGCTCACATTCACCTCCTTTA-3′ (reverse); α-SMA, 5′-GTTCAGTGGTGCCTCTGTCA-3′ (forward) and 5′-ACTGGGACGACATGGAAAAG-3′ (reverse); collagen type 1 alpha 2 (Col1a2): 5′-TAGGCCATTGTGTATGCAGC-3′ (forward) and 5′-ACATGTTCAGCTTTGTGGACC-3′ (reverse); TGF-β: 5′-CGAAGCGGACTACTATGC-3′ (forward) and 5′-GTTGCTCCACACTTGATTT-3′ (reverse); glyceraldehyde-3-phosphate dehydrogenase (GAPDH): 5′-TCACCACCATGGAGAAGGC-3′ (forward); and 5′-GCTAAGCAGTTGGTGGTGCA-3′ (reverse). Reactions (10 μL) were contained 4 µL template cDNA (20 ng), 5 µL KAPA SYBR^®^ FAST qPCR Master Mix (2X), and 1 µL forward/reverse primer mix (6 µM each) (KAPA Biosystems, Boston, MA, USA). Thermal cycling consisted of 15 min at 95 °C, followed by 40 cycles at 95 °C for 15 s and 60 °C for 60 s using the StepOne System (Applied Biosystems, Foster City, CA, USA). The predicted cycle threshold (*C*_t_) values were exported into Excel worksheets for analysis. Comparative *C*_t_ methods were used to determine the gene expression levels relative to that for GAPDH.

### 4.6. Statistical Analyses

Data are shown as mean ± SD. Data from cell studies were evaluated by non-parametric tests. For this purpose, Mann-Whitney *U* test was used to compare 2 independent groups. Kruskal-Wallis followed by Bonferroni posthoc analyses was used to account for multiple testing. Data from animal studies were evaluated by parametric tests. For this purpose, a two-way analysis of variance followed by LSD test was used to make multiple comparisons. The reagent effect and genotypic effect are two independent factors for this analysis. SPSS v20.0 (SPSS Inc., Chicago, IL, USA) was used for analysis. Differences were considered statistically significant at *p* < 0.05.

## References

[1] Schuppan D., Afdhal N.H. (2008). Liver cirrhosis. Lancet.

[2] Bataller R., Brenner D.A. (2005). Liver fibrosis. J. Clin. Investig..

[3] Friedman S.L. (2003). Liver fibrosis—From bench to bedside. J. Hepatol..

[4] Iredale J.P., Thompson A., Henderson N.C. (2013). Extracellular matrix degradation in liver fibrosis: Biochemistry and regulation. Biochim. Biophys. Acta.

[5] Iredale J.P. (2007). Models of liver fibrosis: Exploring the dynamic nature of inflammation and repair in a solid organ. J. Clin. Investig..

[6] Hernandez-Gea V., Friedman S.L. (2011). Pathogenesis of liver fibrosis. Annu. Rev. Pathol..

[7] Forbes S.J., Parola M. (2011). Liver fibrogenic cells. Best Pract. Res. Clin. Gastroenterol..

[8] Friedman S.L. (2008). Hepatic fibrosis—Overview. Toxicology.

[9] Friedman S.L. (2008). Mechanisms of hepatic fibrogenesis. Gastroenterology.

[10] Majumdar A., Curley S.A., Wu X., Brown P., Hwang J.P., Shetty K., Yao Z.X., He A.R., Li S., Katz L. (2012). Hepatic stem cells and transforming growth factor β in hepatocellular carcinoma. Nat. Rev. Gastroenterol. Hepatol..

[11] Lee U.E., Friedman S.L. (2011). Mechanisms of hepatic fibrogenesis. Best Pract. Res. Clin. Gastroenterol..

[12] Inagaki Y., Okazaki I. (2007). Emerging insights into transforming growth factor β Smad signal in hepatic fibrogenesis. Gut.

[13] Fallowfield J.A. (2011). Therapeutic targets in liver fibrosis. Am. J. Physiol. Gastrointest. Liver Physiol..

[14] Yata Y., Gotwals P., Koteliansky V., Rockey D.C. (2002). Dose-dependent inhibition of hepatic fibrosis in mice by a TGF-β soluble receptor: Implications for antifibrotic therapy. Hepatology.

[15] Vanier M.T., Millat G. (2004). Structure and function of the NPC2 protein. Biochim. Biophys. Acta.

[16] Naureckiene S., Sleat D.E., Lackland H., Fensom A., Vanier M.T., Wattiaux R., Jadot M., Lobel P. (2000). Identification of HE1 as the second gene of Niemann-Pick C disease. Science.

[17] Storch J., Xu Z. (2009). Niemann-Pick C2 (NPC2) and intracellular cholesterol trafficking. Biochim. Biophys. Acta.

[18] Ko D.C., Binkley J., Sidow A., Scott M.P. (2003). The integrity of a cholesterol-binding pocket in Niemann-Pick C2 protein is necessary to control lysosome cholesterol levels. Proc. Natl. Acad. Sci. USA.

[19] Sleat D.E., Wiseman J.A., El-Banna M., Price S.M., Verot L., Shen M.M., Tint G.S., Vanier M.T., Walkley S.U., Lobel P. (2004). Genetic evidence for nonredundant functional cooperativity between NPC1 and NPC2 in lipid transport. Proc. Natl. Acad. Sci. USA.

[20] Mukherjee S., Maxfield F.R. (2004). Lipid and cholesterol trafficking in NPC. Biochim. Biophys. Acta.

[21] Liao Y.J., Fang C.C., Yen C.H., Hsu S.M., Wang C.K., Huang S.F., Liang Y.C., Lin Y.Y., Chu Y.T., Arthur Chen Y.M. (2015). Niemann-Pick type C2 protein regulates liver cancer progression via modulating ERK1/2 pathway: Clinicopathological correlations and therapeutical implications. Int. J. Cancer.

[22] Liao Y.J., Lin M.W., Yen C.H., Lin Y.T., Wang C.K., Huang S.F., Chen K.H., Yang C.P., Chen T.L., Hou M.F. (2013). Characterization of Niemann-Pick type C2 protein expression in multiple cancers using a novel NPC2 monoclonal antibody. PLoS ONE.

[23] Ioannou G.N., Morrow O.B., Connole M.L., Lee S.P. (2009). Association between dietary nutrient composition and the incidence of cirrhosis or liver cancer in the united states population. Hepatology.

[24] Ekstedt M., Franzen L.E., Mathiesen U.L., Holmqvist M., Bodemar G., Kechagias S. (2007). Statins in non-alcoholic fatty liver disease and chronically elevated liver enzymes: A histopathological follow-up study. J. Hepatol..

[25] Teratani T., Tomita K., Suzuki T., Oshikawa T., Yokoyama H., Shimamura K., Tominaga S., Hiroi S., Irie R., Okada Y. (2012). A high-cholesterol diet exacerbates liver fibrosis in mice via accumulation of free cholesterol in hepatic stellate cells. Gastroenterology.

[26] Hernandez-Gea V., Ghiassi-Nejad Z., Rozenfeld R., Gordon R., Fiel M.I., Yue Z., Czaja M.J., Friedman S.L. (2012). Autophagy releases lipid that promotes fibrogenesis by activated hepatic stellate cells in mice and in human tissues. Gastroenterology.

[27] Tomita K., Teratani T., Suzuki T., Shimizu M., Sato H., Narimatsu K., Usui S., Furuhashi H., Kimura A., Nishiyama K. (2014). Acyl-CoA:Cholesterol acyltransferase 1 mediates liver fibrosis by regulating free cholesterol accumulation in hepatic stellate cells. J. Hepatol..

[28] Tomita K., Teratani T., Suzuki T., Shimizu M., Sato H., Narimatsu K., Okada Y., Kurihara C., Irie R., Yokoyama H. (2014). Free cholesterol accumulation in hepatic stellate cells: Mechanism of liver fibrosis aggravation in nonalcoholic steatohepatitis in mice. Hepatology.

[29] Kirchhoff C., Osterhoff C., Young L. (1996). Molecular cloning and characterization of HE1, a major secretory protein of the human epididymis. Biol. Reprod..

[30] Maxfield F.R., Tabas I. (2005). Role of cholesterol and lipid organization in disease. Nature.

[31] Repa J.J., Mangelsdorf D.J. (2002). The liver X receptor gene team: Potential new players in atherosclerosis. Nat. Med..

[32] Ikonen E. (2008). Cellular cholesterol trafficking and compartmentalization. Nat. Rev. Mol. Cell Biol..

[33] Ioannou Y.A. (2001). Multidrug permeases and subcellular cholesterol transport. Nat. Rev. Mol. Cell Biol..

[34] Harrison K.D., Miao R.Q., Fernandez-Hernando C., Suarez Y., Davalos A., Sessa W.C. (2009). Nogo-B receptor stabilizes Niemann-Pick Type C2 protein and regulates intracellular cholesterol trafficking. Cell Metab..

[35] Mari M., Caballero F., Colell A., Morales A., Caballeria J., Fernandez A., Enrich C., Fernandez-Checa J.C., Garcia-Ruiz C. (2006). Mitochondrial free cholesterol loading sensitizes to TNF- and FAS-mediated steatohepatitis. Cell Metab..

[36] Simeonova P.P., Gallucci R.M., Hulderman T., Wilson R., Kommineni C., Rao M., Luster M.I. (2001). The role of tumor necrosis factor-α in liver toxicity, inflammation, and fibrosis induced by carbon tetrachloride. Toxicol. Appl. Pharmacol..

[37] Bohan A., Chen W.S., Denson L.A., Held M.A., Boyer J.L. (2003). Tumor necrosis factor α-dependent up-regulation of LRH-1 and MRP3(ABCC3) reduces liver injury in obstructive cholestasis. J. Biol. Chem..

[38] Herrmann J., Gressner A.M., Weiskirchen R. (2007). Immortal hepatic stellate cell lines: Useful tools to study hepatic stellate cell biology and function?. J. Cell. Mol. Med..

